# Predicting the risk of drug-drug interactions in psychiatric hospitals

**DOI:** 10.1192/j.eurpsy.2021.409

**Published:** 2021-08-13

**Authors:** J. Wolff, G. Hefner, C. Normann, K. Kaier, H. Binder, K. Domschke, M. Marschollek, A. Klimke

**Affiliations:** 1 Peter L. Reichertz Institute For Medical Informatics, Medical School Hannover, Hannover, Germany; 2 Forensic Psychiatry, Vitos Clinic, Eltville., Germany; 3 Department Of Psychiatry And Psychotherapy, Medical Center - University of Freiburg, Freiburg, Germany; 4 Institute Of Medical Biometry And Statistics, Medical Center - University of Freiburg, Freiburg, Germany; 5 Department Of Psychiatry And Psychotherapy, Vitos Hochtaunus, Friedrichsdorf, Germany

**Keywords:** Drug Prescriptions, Medication Therapy Management, machine learning, psychiatry

## Abstract

**Introduction:**

The most common medical decision is the prescription of medicines. More than 130 different drugs with proven efficacy are currently available for the treatment of patients with mental disorders.

**Objectives:**

The aim was to use routine data available at a patient’s admission to the hospital to predict polypharmacy and drug-drug interactions (DDI).

**Methods:**

The study used data obtained from a large clinical pharmacovigilance study sponsored by the Innovations Funds of the German Federal Joint Committee. It included all inpatient episodes admitted to eight psychiatric hospitals in Hesse, Germany, over two years. We used gradient boosting to predict respective outcomes. We tested the performance of our final models in unseen patients from another calendar year and separated the study sites used for training from the study sites used for performance testing.

**Results:**

A total of 53,909 episodes were included in the study. The models’ performance, as measured by the area under the ROC, was “excellent” (0.83) and “acceptable” (0.72) compared to common benchmarks for the prediction of polypharmacy and DDI, respectively. Both models were substantially better than a naive prediction based solely on basic diagnostic grouping.

**Conclusions:**

This study has shown that polypharmacy and DDI at a psychiatric hospital can be predicted from routine data at patient admission. These predictions could support an efficient management of benefits and risks of hospital prescriptions, for instance by including pharmaceutical supervision early after admission for patients at risk before pharmacological treatment is established

**Disclosure:**

This work was supported by the Innovations Funds of the German Federal Joint Committee (grant number: 01VSF16009). The funding body played no role in the design of the study and collection, analysis, and interpretation of data and in writing the manuscrip

